# Profiling of different pancreatic cancer cells used as models for metastatic behaviour shows large variation in their *N*-glycosylation

**DOI:** 10.1038/s41598-017-16811-6

**Published:** 2017-11-30

**Authors:** Stephanie Holst, Ana I. Belo, Elisa Giovannetti, Irma van Die, Manfred Wuhrer

**Affiliations:** 1 Center for Proteomics and Metabolomics, Leiden University Medical Center, Leiden, The Netherlands; 2 Department of Molecular Cell Biology and Immunology, VU University Medical Center, Amsterdam, The Netherlands; 3 Department of Medical Oncology, VU University Medical Center, Amsterdam, The Netherlands; 4 Cancer Pharmacology Lab, AIRC Start-Up Unit, University of Pisa, Pisa, Italy

## Abstract

To characterise pancreatic cancer cells from different sources which are used as model systems to study the metastatic behaviour in pancreatic ductal adenocarcinoma (PDAC), we compared the *N*-glycan imprint of four PDAC cells which were previously shown to differ in their galectin-4 expression and metastatic potential *in vivo*. Next to the sister cell lines Pa-Tu-8988S and Pa-Tu-8988T, which were isolated from the same liver metastasis of a PDAC, this included two primary PDAC cell cultures, PDAC1 and PDAC2. Additionally, we extended the *N*-glycan profiling to a normal, immortalized pancreatic duct cell line. Our results revealed major differences in the *N*-glycosylation of the different PDAC cells as well as compared to the control cell line, suggesting changes of the *N*-glycosylation in PDAC. The *N-*glycan profiles of the PDAC cells, however, differed vastly as well and demonstrate the diversity of PDAC model systems, which ultimately affects the interpretation of functional studies. The results from this study form the basis for further biological evaluation of the role of protein glycosylation in PDAC and highlight that conclusions from one cell line cannot be generalised, but should be regarded in the context of the corresponding phenotype.

## Introduction

Although death rates for many cancers have decreased by more than 20% in the last 30 years, the incidence and mortality for pancreatic cancer is still rising^1,2^. Asymptomatic early stages as well as lack of reliable early diagnosis biomarkers hinder the detection of pancreatic cancer before spreading to other organs, and contribute to its high mortality^3,4^. Once metastases have occurred, most pancreatic cancers are resistant to chemotherapy and only 15–20% of the patients benefit from surgery, resulting in a 5-year-survival below 5%^2,5^. For pancreatic ductal adenocarcinoma (PDAC), which accounts for about 90% of pancreatic cancers, a very early formation of metastases is typical, hence increasing malignancy and therapy resistance considerably^6^. For these reasons, understanding the biology and mechanisms underlying PDAC progression and metastasis formation is essential to improve early detection and treatment.

Several studies identified genetic alterations in main signalling pathways with high occurrence of mutations in the oncogene *K-ras*, and in the tumour-suppressor genes *TP53, SMAD4*, and *CDKN2A* ^2,7^. However, multiple pathogenic pathways leading to PDAC have been proposed, also as a result of the identification of different precursor lesions^7,8^.

On a glycomic level, several carbohydrate antigens such as CA-19–9, carcinoembryonic antigen, CA242, or combinations of those are used for pancreatic cancer diagnosis or monitoring^9,10^. Recently, fucosylated haptoglobin, but also glycosylated isoforms of α-1-acid glycoprotein, α-antitrypsin, and sialylated (*Sambucus nigra* lectin-responsive) α-1-β-glycoprotein have been explored for early detection of PDAC^11–14^. With regard to understanding PDAC biology and metastasis, characterisation of the aberrant glycosylation on cancer cells is a first step and can identify potential biomarkers, while *in vitro* and *in vivo* studies as well as manipulations of the glyco-phenotype can provide information on mechanism and therapy targets. However, the cell line model systems need to be well characterised in order to choose the model most reflecting the cancer phenotype and to interpret results correctly.

We previously described the effect of galectin-4 expression of two closely related PDAC cell lines (the established sister cell lines Pa-Tu-8988S (PaTu-S) and Pa-Tu-8988T (PaTu-T)) on their metastatic behaviou^15,16^. While the two sister cell lines PaTu-S and PaTu-T were derived from the same liver metastasis of a patient with PDAC, thereby having the same genetic background, their metastatic behaviour differed vastly *in vitro* and *in vivo* in Danio rerio (zebrafish)^15,16^. Since galectin-4 is a glycan binding protein, and differentially binds the two cell lines, we hypothesised that the surface glycosylation would differ between PaTu-S and PaTu-T. Therefore, we characterised the *N-*glycome of these cell lines in order to (i) investigate if the *N*-glycan phenotype differs between the tumour-like PaTu-S and the metastatic PaTu-T, and (ii) characterise the *N*-glycan phenotype for future *in vitro* and *in vivo* studies using PaTu-S and PaTu-T as model systems. We expanded the characterisation to two primary cultures (PDAC1 and PDAC2), which as well showed different galectin-4 expression and metastatic behaviour^15,17^, and included the comparison to a normal, immortalised pancreatic duct cell line (hTERT-HPNE). Hitherto, only few studies have been performed to comprehensively characterise the glycosylation of cell line model systems using mass spectrometry^18,19^ and, importantly, evaluating their potential as model system by comparing cell line glycosylation profiles with those of tissues^20^. Especially in biopharmaceutical production, the selection of the right production system gained importance^21^, while for functional studies this awareness is still scarce.

Our results show that the investigated cells differ vastly in their *N-*glycome, emphasising the importance of the phenotypic characterisation of cell lines for the interpretation of *in vitro* or *in vivo* experiments. Interestingly, the tumour-like PaTu-S revealed the most deviating complex-type *N-*glycan features as compared to the immortalized normal pancreatic duct cell line, while the metastatic sister cell line PaTu-T showed most similar characteristics to this normal duct cell line.

## Results and Discussion

### Cell characteristics

Two PDAC cell lines and two primary PDAC cell cultures, which were characterised by different metastatic behaviour *in vitro* and *in vivo*, were characterised with regard to their *N-*glycan profile and compared to an immortalised, normal pancreatic duct cell line. While comparison of PaTu-S and PaTu-T is of particular interest due to their shared genetic background, the early-passage primary cultures PDAC1 and PDAC2 may better represent the genetic characteristics of the original tumour. As described before, PaTu-S showed a primary, tumour-like behaviour with an epithelial phenotype, whereas PaTu-T revealed a mesenchymal phenotype with high migratory and metastatic capacity *in vitro* and *in vivo* in zebrafish^15,16^. The primary cell cultures PDAC1 and PDAC2 were isolated from two different patients with PDAC in the same stage based on the pathological tumour-node-metastasis (pTNM) staging system. However, PDAC1 was derived from a male and PDAC2 from a female with a shorter survival time (8.5 months in PDAC2 vs. 21.4 months in PDAC1)^22^. In culture, PDAC2 revealed a less cohesive pattern of growth, suggesting a more mesenchymal phenotype as compared to PDAC1. In mouse models, PDAC1 showed a significantly lower migratory and invasive potential as compared to PDAC2^17^, which was comparable to the behaviour of PaTu-S and PaTu-T in zebrafish, respectively. In contrast, both PDAC1 and PDAC2 showed a dramatically more aggressive behaviour in the zebrafish model as compared to PaTu-S and PaTu-T. For PDAC1 more than 23% of the fish were dying within 48 h of the experiment and for PDAC2 44% (vs. less than 15% in both PaTu-cells; unpublished data). Furthermore, for both PDAC cells a strong occurrence of brain metastases was observed in zebrafish (≥20% for both PDAC cell cultures vs. <10% for both PaTu cell lines; unpublished data).

### Mass spectrometric profiling and characterisation of *N-*glycans from pancreatic cancer cell lines

To determine how the pancreatic cancer cells PaTu-S, PaTu-T, PDAC1, and PDAC2 and the normal pancreatic duct cell line hTERT-HPNE differ in their *N-*glycosylation, *N-*glycans were released from the proteins using a PVDF-membrane-based 96-well plate protocol^18^. Prior to MALDI-TOF-MS analysis, *N-*acetylneuraminic acids (NeuAc) were subjected to linkage-specific derivatisation using a recently developed protocol allowing structural stabilisation of the sialic acid residues and the mass spectrometric distinction between α2,3 and α2,6-linked species on the basis of mass shifts induced by ethyl esterification (α2,6-linkage) and lactonisation (α2,3-linkage)^23^. Compositions of detected glycans were confirmed by fragmentation analysis using MALDI-TOF/TOF-MS/MS as well as additional reversed-phase liquid chromatography (RP-LC)-MS/MS of 2-aminobenzoic acid (AA)-labelled glycan pools. A list of compositions with averaged abundance per sample and fragmentation information is given in Supplemental Table S1. Glycan features were assigned on the basis of the fragmentation spectra, signal patterns evident from the profiling spectra and general glycobiological knowledge. Information on glycosidic linkage positions was obtained solely for NeuAc residues on the basis of mass shifts induced by the derivatisation^23^.

**Figure 1:**
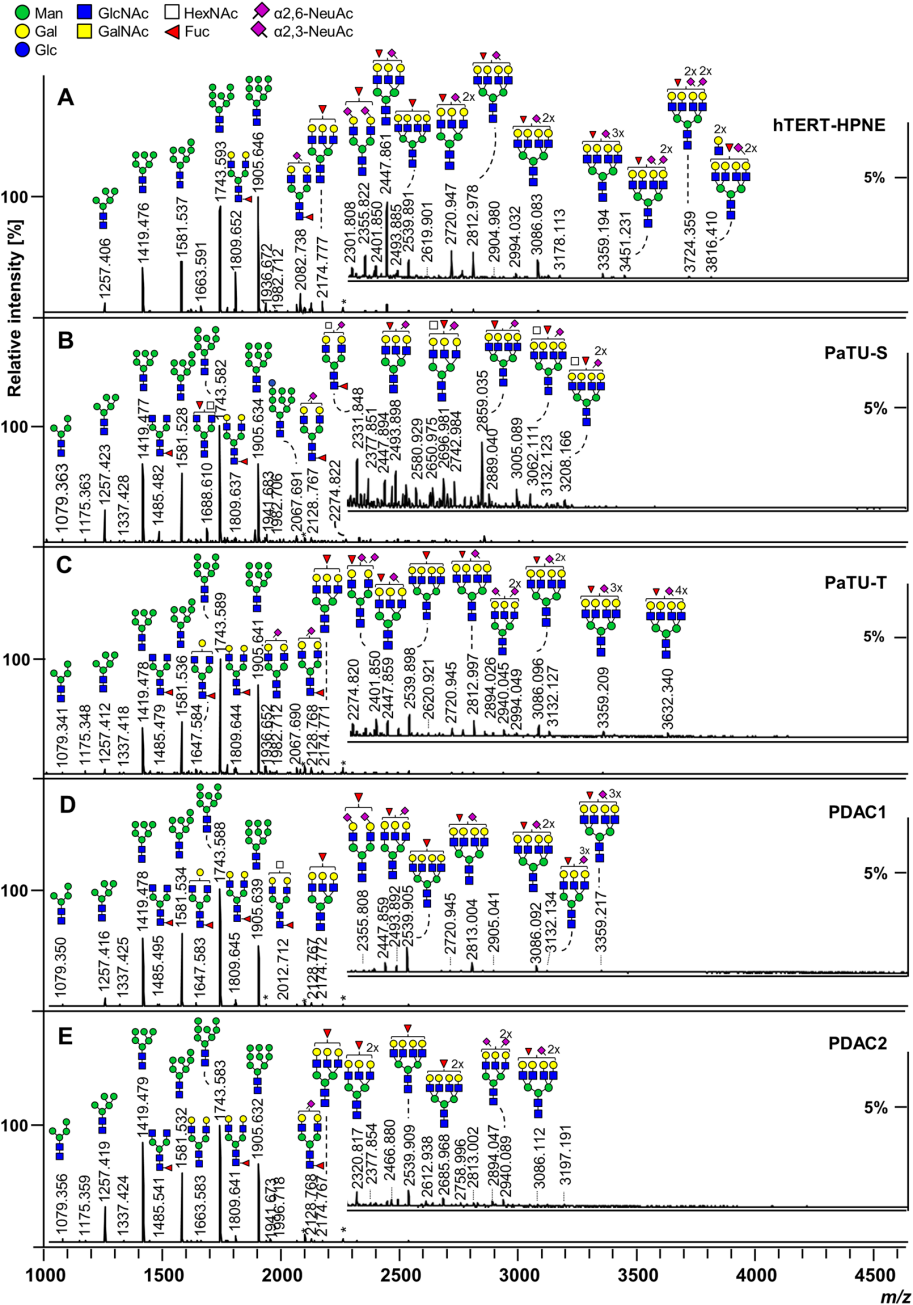
Representative positive ion mode MALDI-TOF-MS spectra of released and derivatized *N-*glycans. Exemplary mass spectra of (**A**) Pancreatic duct cell line hTERT-HPNE, **(B)** Pancreatic duct adenocarcinoma (PDAC) sister cell lines PaTu-S and (**C**) PaTu-T, as well as the primary PDAC cell cultures **(D)** PDAC1, and (**E**) PDAC2 are shown. Major glycan peaks are annotated. Symbolic glycan depictions represent compositions and the presence of structural isomers cannot be excluded. Glc = glucose; Gal = galactose; Man = mannose; GlcNAc = *N-*acetylglucosamine; GalNAc = *N-*acetylgalactosamine; Fuc = deoxyhexose, fucose; NeuAc = *N-*acetylneuraminic acid; *reducing end adduct.

**Figure 2:**
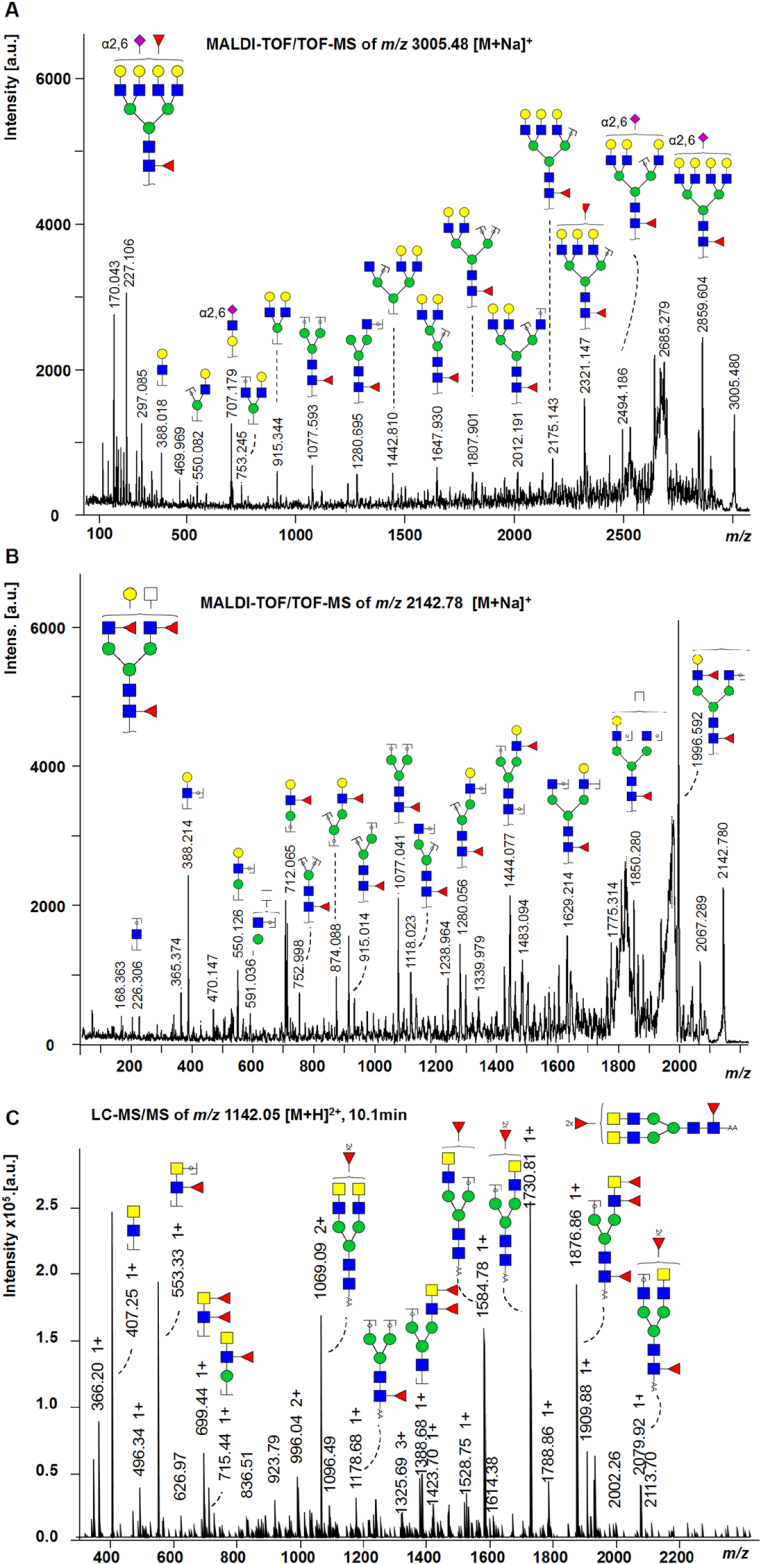
Fragmentation spectra. (**A)** MALDI-TOF/TOF-MS/MS fragmentation spectrum of the *N*-glycan Hex7HexNAc6Fuc2(α2,6)NeuAc1 with *m/z* 3005.48 [M + Na]^+^. The fragment ion at *m/z* 707.2 [M + Na]^+^ is indicative for Hex1HexNAc1(α2,6)NeuAc1. The mass shift of + 28 Da from a non-modified *N*-acetylneuraminic acid to an ethyl esterified *N*-acetylneuraminic acid indicates α2,6-linkage. The position of the α2,6NeuAc as well as the antenna fucose cannot be identified. (**B**) MALDI-TOF/TOF-MS/MS fragmentation spectrum of the *N*-glycan Hex4HexNAc5Fuc3 with *m/z* 2142.78 [M + Na]^+^. Fragment ions for antenna-fucosylation (*m/z* 712.1 [M + Na]^+^, *m/z* 874.1 [M + Na]^+^) as well as core-fucosylation (*m/z* 1077.0 [M + Na]^+^) were identified. **(C)** LC-MS/MS fragmentation spectrum of the *N*-glycan Hex3 HexNAc6Fuc3 with *m/z* 1142.05 [M + H]^2+^. Indicative fragment ions at *m/z* 407 [M + H]^+^ (HexNAc2) and *m/z* 553 [M + H]^+^ (HexNAc2dHex1) show the presence of LacdiNAc structures. Annotation was performed in GlycoWorkbench 2.1 stable build 146 (http://www.eurocarbdb.org/) using the Glyco-Peakfinder tool (http://www.eurocarbdb.org/ms-tools/). The presence of structural isomers cannot be excluded. Hex = hexose; blue circle = Glc, glucose; yellow circle = Gal, galactose; green circle = Man, mannose; blue square = GlcNAc, *N-*acetylglucosamine; yellow square = GalNAc, *N*-acetylgalactosamine; white square = HexNAc, *N*-acetylhexosamine; red triangle = Fuc, deoxyhexose, fucose; purple diamond = NeuAc, *N-*acetylneuraminic acid; *reducing end adduct.

**Figure 3:**
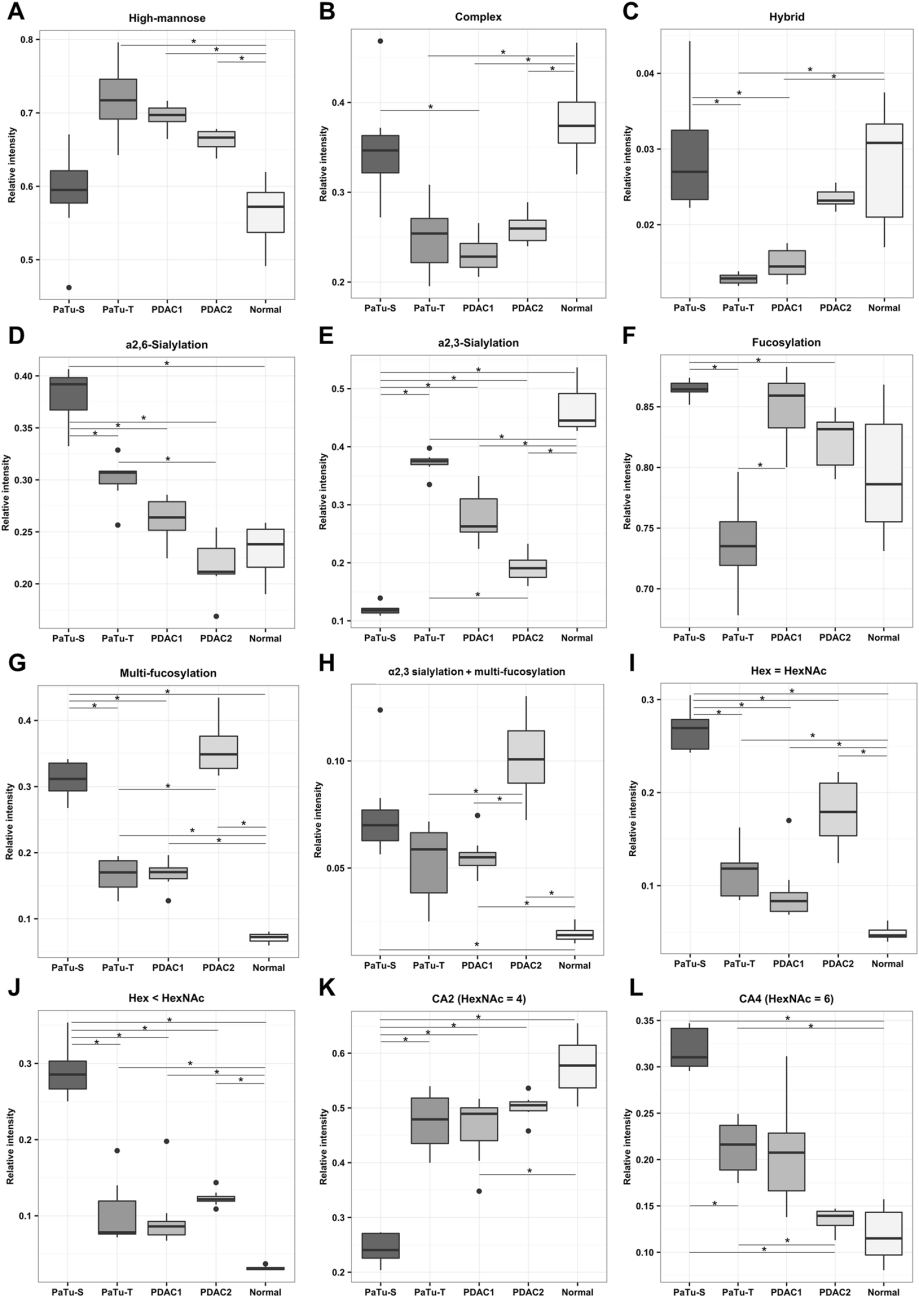
Analysis of structural *N-*glycan classes. Derived traits were calculated and averaged per cell line for the biological replicates from mass spectrometry analysis. Boxplots are illustrated with median and interquartile range. **(A)** Total high-mannose type content, **(B)** Total complex-type *N-*glycans, **(C)** Total hybrid type structures. Following derived traits were calculated including exclusively *N*-glycans of the complex type: **(D)** α2,6-Sialylation, **(E)** α2,3-Sialylation, **(F)** Fucosylation, **(G)** Multi-fucosylation defined as the presence of more than one fucose, representative for antenna-fucosylation, **(H)** Multi-fucosylation of α2,3-sialylated *N*-glycans, indicative for sialyl Lewis antigens, **(I)** *N-*glycans featuring an equal number of *N-*acetylhexosamines (HexNAc) compared to hexoses (Hex), **(J)** *N-*glycans featuring smaller number of Hex than HexNAc, **(K)** *N*-glycans with HexNAc = 4, indicative for di-antennary *N*-glycans, **(L)** *N*-glycans with HexNAc = 6, indicative for tetra-antennary *N*-glycans as well as LacNAc-repeats, bisecting GlcNAc, or GalNAcs additions. Traits were tested for significant differences between the samples and *p-values* are given in Supplemental Table S7.

Representative *N-*glycan profile spectra with the main peaks annotated are shown in Fig. 1 for all five PDAC cells and exemplary fragmentation spectra are depicted in Fig. 2. The *N-*glycan profiles of all investigated cells ranged from *m*/*z* 1000 to approximately *m/z* 4000. Profiles were dominated by high-mannose-type *N-*glycans for all cell lines yet to different extent. The normal pancreatic duct cell line hTERT-HPNE and PaTu-S showed comparable levels of high-mannose type *N-*glycans (ø 56% and ø 59%), while the more metastatic cells PaTu-T, PDAC1, and PDAC2 showed elevated levels of ø 66–72% high-mannose *N*-glycans with highest abundance in PaTu-T (Supplemental Table S2, Fig. 3A). Accordingly, Park *et al*. described a higher content of high-mannose type *N*-glycans in a pancreatic cancer cell line from liver metastasis as compared to cell lines from the pancreas duct head and tail regions as well as a normal pancreatic cell line^24^. Also in other cancers, such as colorectal cancer, elevated levels of high-mannose type *N*-glycans were found^25,26^. Various complex-type *N-*glycans with and without sialylation and/or fucosylation were identified and showed accordingly a trend opposite to that of high-mannose type glycans as an effect of total area normalisation (Fig. 3B). Hybrid-type structures were only low abundant in all investigated cells with PaTu-S showing highest (ø 3%) and PaTu-T lowest (ø 1%) levels (Supplemental Table S2, Fig. 3C).

### Pronounced differences in complex type *N-*glycan features between the cells

Derived glycan traits for features like sialylation, fucosylation, antennarity, and others were calculated exclusively for complex-type *N-*glycans, thereby re-scaling the data to 100% with exclusion of relative intensities from high-mannose or hybrid type *N*-glycans. This brings the advantage that true complex-type *N*-glycan changes were observed and alterations seen are not a result of differences in overall abundance of complex-type *N-*glycans.

**Figure 4:**
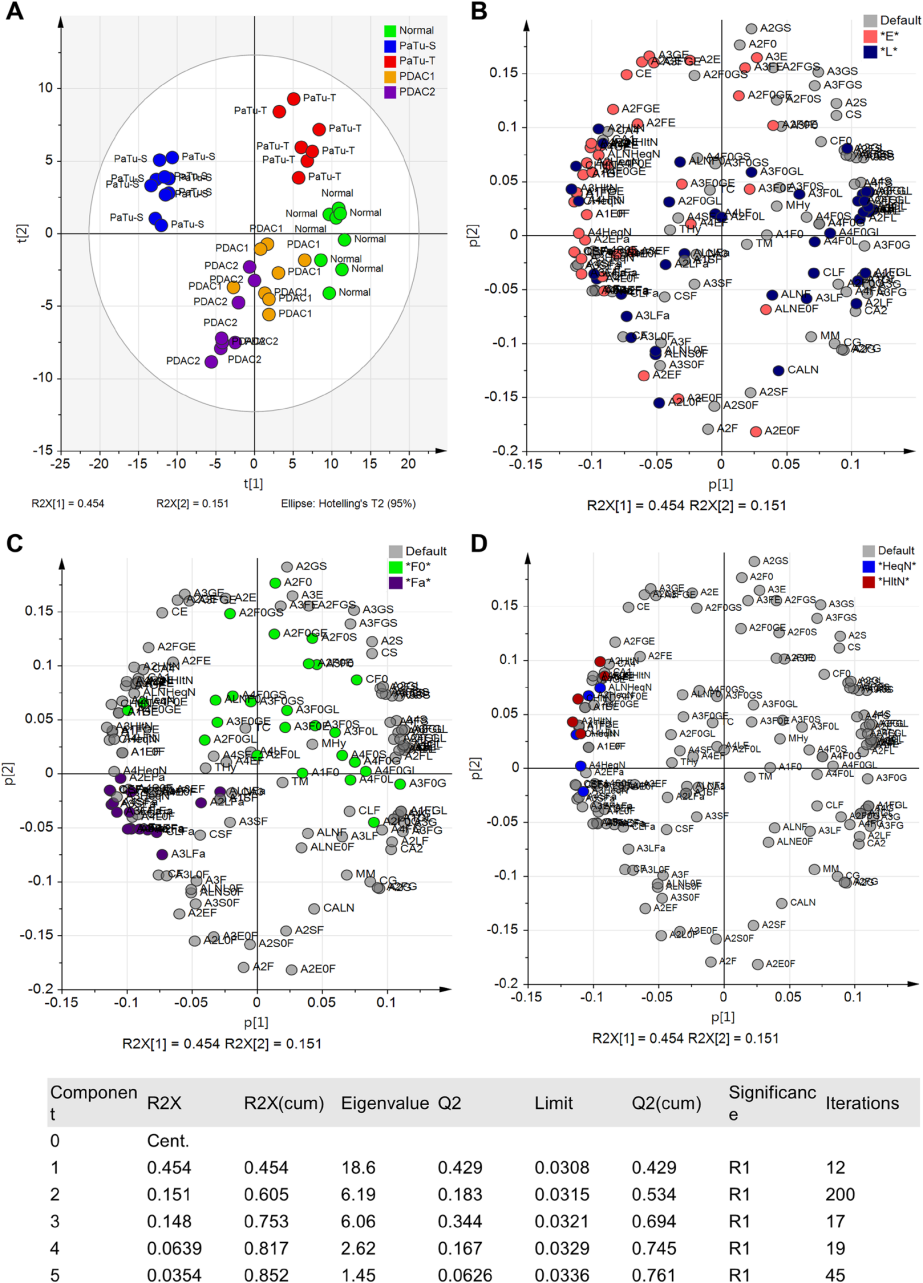
Principal Component Analysis (PCA). The PCA resulted in five principal components (PCs) explaining 85.2% of variation in the data (R2Xcum) with a very good prediction power (Q2cum) of 76%. Unit variance (UV)-scaling was applied to the data and validation of the model was performed by internal cross-validation (CV) based on biological replicates (n = 8) as CV groups. **(A)** Score plot of PC1 (45.5%) vs PC2 (15.1%) demonstrating the largest separation between PaTu-S and the normal cell line on PC1 and between the two cell lines PaTu-S and PaTu-T versus the two primary cell cultures PDAC1 and PDAC2 on PC2. (**B**) Corresponding loading plot of PC1 vs. PC2 illustrating the derived *N*-glycan traits on which the PCA model is build. Coloring the loadings according to glycan features, here α2,6-sialylation (**E**), blue) vs. α2,3-sialylation (L, rose) vs. non-sialylated (default, grey), was used in order to facilitate the identification of differences between the samples. **(C)** Loading plot as in B, colored according to fucosylation with non-fucosylated (F0, green) vs. multi-fucosylated (Fa, purple). (**D**) Loading plot as B, colored according to the ratio of the number of hexoses (Hex, H) to the amount of *N*-acetylhexosamines (HexNAc, N) with H equal to N (HeqN, blue) vs. H smaller than N (HltN, red). The table provides the statistic of the model.

To elucidate differences of the *N-*glycan profiles between the cell lines and to extract possible discriminators, multivariate statistical analysis was performed. A principal component analysis (PCA) model was built on the relative abundance of derived traits resulting in a model with five principal components, covering 85% of variation (R^2^Xcum) in the data with a very good prediction power (Q^2^cum) of 76% (Fig. 4).

#### Sialylation

**Figure 5:**
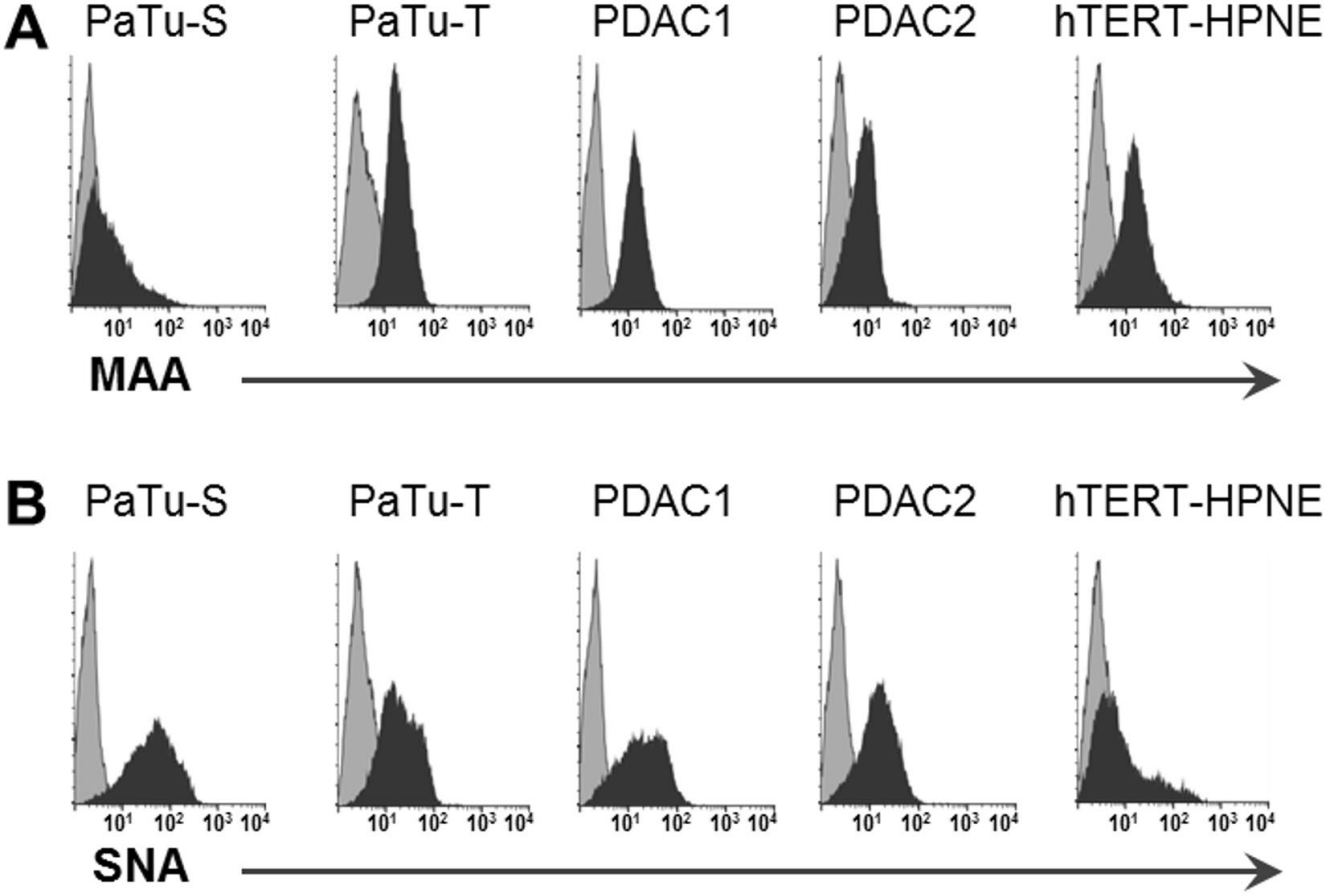
Flow cytometry binding assay with plant lectins. The avidity of binding of plant lectins **(A)** *Maackia amurensis agglutinin* (MAA) and **(B)** *Sambucus nigra agglutinin* (SNA) to PaTu-S, PaTu-T, PDAC1, PDAC2, and hTERT-HPNE was determined. Overlay histograms of representative experiments from at least three independent experiments are shown. Dark grey field: staining with the antibody against the respective structure by means of fluorescent intensity; light grey field: background staining with secondary antibodies. Averaged mean fluorescence intensities (MFI) are given in Supplemental Table S3.

The score plot of principal components (PC) one and two showed most pronounced separation between PaTu-S and the control cell line on PC1 (45%; Fig. 4A) which could be attributed to differences in sialylation based on the corresponding loading plot (Fig. 4B). The control cell line hTERT-HPNE seemed to be characterised by higher levels of α2,3-sialylation (blue, Fig. 4B), whereas α2,6-sialylated *N-*glycans (red, Fig. 4B) appeared more abundant in PaTu-S. Cell cultures PDAC1 and PDAC2 located around PC1, suggesting a mixed sialylation profile, while PaTu-T clustered more closely to the normal cell line, thereby a higher content of α2,3-sialylation was expected. In accordance, derived trait calculations showed that sialylation in α2,6-linkage was most abundant in PaTu-S (ø 38%) and decreased in PaTu-T (ø 30%), PDAC1 (ø 26%), hTERT-HPNE (ø 23%), and PDAC2 (ø 22%; Supplemental Table S2, Fig. 3D). The fragmentation spectrum in Fig. 2A shows an indicative fragment ion at *m/z* 707.2 corresponding to [Hex1HexNAc1NeuAc(2,6)1 + Na]^+^ (Hex, H = hexose; HexNAc, N = *N-*acetylhexosamine; NeuAc, S = *N-*acetylneuraminic acid, sialic acid) and confirmed the presence of an ethyl esterified α2,6-sialylated antenna. In contrast, the relative abundance of α2,3-sialylation was highest in normal hTERT-HPNE cells (ø 47%), followed by PaTu-T (ø 37%), PDAC1 (ø 28%), PDAC2 (ø 19%) and was lowest in PaTu-S (12%; Supplemental Table S2, Fig. 3E). This is partially in accordance with literature since increased α2,3-sialylation was associated with metastasis via sialyl Lewis interactions with E-selectin^27^, and increased levels were observed for the more metastatic cell line PaTu-T as compared to the tumour-like cell line PaTu-S. Moreover, transfection of pancreatic cancer cell lines with α2,3-sialyltransferase III increased expression of sialyl Lewis X combined with a lower α2,6-sialylation on α2β1-integrin and correlated with increased invasive behaviour as well as reduced cell-cell aggregation^28^. The high levels of α2,3-sialylation in the control pancreatic duct cell line remain, however, unexpected and also overall sialylation (S) was highest in hTERT-HPNE cells (ø 61%), whereas lowest in PDAC2 (39%; Supplemental Table S2). Low sialylation has been described by Park *et al*. on permethylated *N*-glycans from a pancreatic cancer cell line derived from a liver metastasis, but no distinction between α2,3-sialylation and α2,6-sialylation was made^24^. To determine the relative expression of sialic acids at the surface of the different cells, flow cytometry was performed using plant lectins that bind to terminally sialylated glycans independent of the glycan class (*N*-, *O*- and glycolipid-glycans): *Maackia amurensis agglutinin* (MAA; α2,3-sialylation; Fig. 5A) and *Sambucus nigra agglutinin* (SNA; α2,6-sialylation; Fig. 5B). The binding of MAA and SNA lectins correlated well with the results obtained by mass spectrometry on *N*-glycans and mean fluorescence intensities (MFI) are summarized in Supplemental Table S3. In line with *N*-glycan derived traits for sialylation, the relative abundance of α2,3-sialylation based on MAA-binding (Fig. 5A) was highest in PaTu-T (ø MFI 17.2) followed by normal hTERT-HPNE cells (ø MFI 15.6), PDAC1 (ø MFI 12.9), and was lowest in PaTu-S (ø MFI 8.4) and PDAC2 (ø MFI 5.9). In contrast, and in conformance with the MS results, sialylation in α2,6-linkage as detected by SNA binding (Fig. 5B) was most abundant in PaTu-S (ø MFI 63.1) and decreased with PaTu-T (ø MFI 25.8) and PDAC1 (ø MFI 25.8) which showed comparable levels of α2,6-sialylation. PDAC2 (ø MFI 17.8) and cells of the normal immortalised cell line hTERT-HPNE showed least binding to SNA (ø MFI 18.9). The confirmation of the results obtained by mass spectrometry of *N*-glycans after linkage-specific sialic acid derivatisation with lectin-binding indicates that observed changes on *N*-glycans likely also occur on *O*-glycans and/or glycolipid glycans. Alternatively, one may speculate that *N*-glycans are the dominant glycan class on these cells largely determining lectin binding.

#### Fucosylation

On PC2 (15%) the main separation was between the two cell lines PaTu-S and PaTu-T versus the two primary cell cultures PDAC1 and PDAC2 (Fig. 4A). Investigations of the loading plot revealed differences in non- versus multi-fucosylated *N-*glycans contributing to the separation partly on PC1 as well as PC2. Based on the loading plot, multi-fucosylation (purple, Fig. 4C) was mainly expected in PDAC2 cells, while highest abundance of non-fucosylated *N-*glycans (green, Fig. 4C) was predicted for PaTu-T. Derived trait analysis confirmed this observation and revealed pronounced differences in fucosylation (F) and multi-fucosylation (number of fucoses ≥ 2), the latter indicative for antenna-fucosylation (Fa). The PaTu-S and PDAC1 cells were characterised with highest overall fucosylation (ø 87% and ø 85%), whereas PDAC2 showed in accordance with the PCA analysis most multi-fucosylated complex *N-*glycans (ø 36%; Supplemental Table S2, Fig. 3F,G). Fragmentation analysis confirmed antenna fucosylated *N-*glycans and an exemplary MALDI-TOF/TOF-MS/MS spectrum for the *N-*glycan with composition Hex4HexNAc5dHex3 (dHex = fucose) at *m*/*z* 2142.78 [M + Na]^+^ is shown in Fig. 2B. Fucosylation was lowest in PaTu-T cells (ø 74% total fucosylation and ø 17% multi-fucosylation), which is in accordance with results from the above mentioned study by Park *et al*. for a liver metastatic pancreatic cancer cell line^24^, but also in our control pancreatic cell line fucosylation was very low (ø 80% total fucosylation and ø 7% multi-fucosylation; Supplemental Table S2, Fig. 3F,G). Interestingly, increased fucosylation was previously associated with pancreatic cancer stem cell-like phenotypes^29^.

#### (Sialyl) Lewis antigens

A similar pattern as seen for multi-fucosylated structures, indicative for Lewis antigens, was also observed for α2,3-sialylated *N-*glycans carrying additional fucoses (n ≥ 2, multi-fucosylation), a derived trait representative for sialyl Lewis antigens. Highest abundance from MS-based analysis was observed for PDAC2 (ø 10%), followed by PaTu-S (ø 8%), PaTu-T (ø 5%), and PDAC1 (ø 6%), whereas hTERT-HPNE showed lowest abundance (ø 2%; Supplemental Table S2, Fig. 3H).

Since the exact position of fucoses and sialic acids could, however, not be determined by tandem MS experiments, flow cytometric analyses with antibodies against (sialyl) Lewis antigens were performed (Supplemental Table S3) and confirmed their presence. Like the above described lectins, these antibodies identify the expression of the antigens on the entire cell surface, thereby not being specific for *N-*glycans only, but also detecting *O-*glycans and glycans on glycolipids.

**Figure 6:**
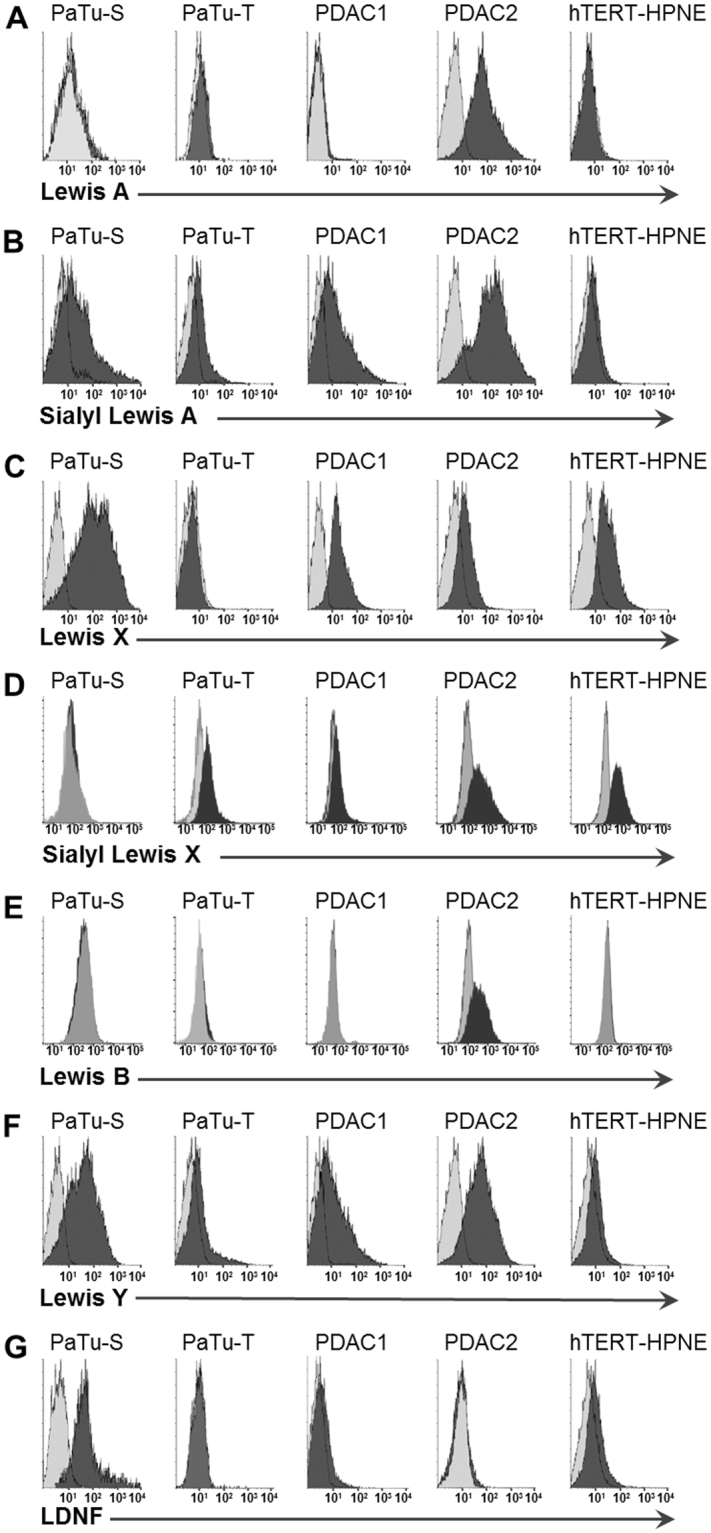
Flow cytometry binding assay with monoclonal antibodies. Binding of antibodies recognizing **(A)** Lewis A, **(B)** sialyl Lewis A (CA 19-9), **(C)** Lewis X, **(D)** sialyl Lewis X **(E)** Lewis B, **(F)** Lewis Y and (**G)** LDNF to PaTu-S, PaTu-T, PDAC1, PDAC2, and hTERT-HPNE was investigated. Overlay histograms of representative experiments from at least three independent experiments are shown. Dark grey field: staining with the antibody against the respective structure by means of fluorescent intensity; light grey field: background staining with secondary antibodies. Averaged mean fluorescence intensities (MFI) are given in Supplemental Table S3.

The flow cytometry experiments confirmed and refined MS-based results and revealed that multi-fucosylation on PDAC2 is mainly attributed to presence of Lewis A (ø MFI 147.3; Fig. 6A), Lewis B (ø MFI 414.7; Fig. 6E), and Lewis Y (ø MFI 75.9; Fig. 6F), but also Lewis X was expressed (ø MFI 38.8, Fig. 6C). Strikingly, PDAC2 showed even higher binding of sialyl Lewis A (ø MFI 463.5, Fig. 6B) and sialyl Lewis X antibodies (ø MFI 752.3, Fig. 6D), which matches the results for the derived trait representative for sialyl Lewis antigens but is somewhat surprising when looking at MAA-binding and the relative low abundance of α2,3-sialylation. Sialyl Lewis antigens have been reported to promote extravasation and metastasis^27,28^, which is in line with the strongly invasive character of PDAC2.

Notably, the immortalised normal cell line hTERT-HPNE showed highest binding of anti-sialyl Lewis X antibodies (ø MFI 780.7, Fig. 6D) which is in accordance with observed high levels of α2,3-sialylation, but is in contrast to reports associating sialyl Lewis X strongly with metastasis in various cancers^30^. The latter might result from studies not including control cell lines, thereby missing important comparisons. However, also in tissues of normal pancreas the absence or reduced expression of sialyl Lewis antigens has been described using the same anti-sialyl Lewis X antibody clone FH6^31,32^. Anti-sialyl Lewis X antibody clone FH6 recognises various forms of the sialyl Lewis epitope, including the monomeric, dimeric and sulphated variant, but importantly only *N*-acetylneuraminic acid and not *N*-glycolylneuraminic acid containing epitopes^33^. Next to sialyl Lewis X, also expression of Lewis X (ø MFI 43.4, Fig. 6C) in hTERT-HPNE cells was comparable to PDAC2. In contrast, hTERT-HPNE cells showed as expected only low binding to antibodies against sialyl Lewis A (CA19-9; ø MFI 6.5, Fig. 6B), which is used as diagnostic marker for pancreatic cancer. Also binding to the non-sialylated antigens Lewis A (not detected, Fig. 6A), Lewis B (ø MFI 14.7, Fig. 6E) and Lewis Y (ø MFI 5.8, Fig. 6F) were low or absent. These partially contradicting results question the use of hTERT-HPNE as a normal control cell line. Though this immortalized cell line was found positive for telomerase and Nestin expression, while no cancer-associated changes (diploid and expressing wild type p16INK4a, p53, K-Ras) were detected^34^, the immortalization through stable transfection with human telomerase reverse transcriptase (hTERT) can influence the cells’ characteristics. Feldman *et al*. reviewed critically the use of *in vitro* models in pancreatic cancer research and questioned the genuinely ‘normal’ character of hTERT-HPNE^35^ and also Maqsood *et al*. discuss the advantages and challenges of immortalization of cell lines^36^, while others described human cells immortalized with hTERT as closest resemblance to primary human cells as *in vitro* system without turning the cells into tumor cells as seen for immortalization with viral oncogenes^37^.

PaTu-T, likewise a metastatic PDAC cell line, shared high levels of α2,3-sialylation with the normal cell line and showed also relatively high expression of sialyl Lewis X (ø MFI 144.8, Fig. 6D) and very low expression of sialyl Lewis A (ø MFI 12.7, Fig. 6B). Also, the non-sialylated Lewis antigens were only very low-abundant (ø MFI 1.1–10.1, Fig. 6A, C, E, F) which is in accordance with mass spectrometric results revealing lowest fucosylation in PaTu-T. In this study, the more mesenchymal cells PDAC2 and especially PaTu-T show similarities regarding their glycosylation phenotype to that of the normal pancreatic duct cell line, suggesting that these cells partially mimic the normal cell line - which may promote immune invasion and metastasizing, but also raising concerns as to the normal character of the control cell line.

PDAC1 shared high expression of sialyl Lewis X (ø MFI 239.7, Fig. 6D) with the previously described cell cultures and also showed high expression of sialyl Lewis A (ø MFI 56.3, Fig. 6B), whereas the non-sialylated Lewis antigens were moderately (Lewis X ø MFI 23.5, Fig. 6C; Lewis Y ø MFI 29.0, Fig. 6F) or not expressed (Lewis A, Fig. 6A; Lewis B, Fig. 6E).

PaTu-S, in contrast, is the most tumour-like cell line and was, as PDAC2, characterised by high fucosylation levels, especially multi-fucosylation, and low α2,3-sialylation. Flow cytometry experiments revealed highest expression of Lewis X (ø MFI 154.7, Fig. 6C) and Lewis Y (ø MFI 84.0, Fig. 6F) in PaTu-S amongst all tested cell lines. Lewis B, on the other hand was not detected (Fig. 6E) and Lewis A was only very low (ø MFI 9.1, Fig. 6A). Sialyl Lewis A was very high in PaTu-S (ø MFI 181.8, Fig. 6B), similar to PDAC2, while sialyl Lewis X was not detected (Fig. 6D).

In line with these results, we previously showed enhanced levels of FUT1 and FUT2 as well as the resulting Lewis Y and blood group antigens in PaTu-S compared to PaTu-T^38^. Additionally, hypoxia inducible factor 1 alpha (HIF1 α) was found higher expressed in PaTu-T compared to PaTu-S and was identified to suppress FUT1/2 expression in pancreatic and colon cancer cell lines, leading to the reduced expression of the resulting epitopes^38^. Aubert *et al*. hypothesized that FUT1 is competing with α2,3-sialyltransferases for modification of type 2 chains, thereby skewing the glycan profile to sialyl Lewis epitopes through reduced FUT1 expression during pancreatic cancer progression^39^. Their results showed reduced α1,2-fucosyltransferase activity in pancreatic cancer cell lines in comparison to a normal pancreatic cell line^39^. Furthermore, restoration of FUT1 in the pancreatic cancer cell line BxPC-3 led to reduced adhesive and metastatic properties, and was associated with a reduction of sialyl Lewis A and sialyl Lewis X expression^39^. Conversely, our results showed low expression for Lewis B and Y in the normal cell line, whereas in PDAC2 we observed high levels of Lewis B and Y together with high levels of (sialyl) Lewis A and X. This is in contrast to reports which identified Lewis Y and B epitopes mainly on normal pancreatic tissues, whereas Lewis X, sialyl Lewis X, and sialyl Lewis A were detected in pancreatic cancer tissues^32^. Interestingly, in colon cancer cell lines, Lewis Y expression was positively associated with sialyl Lewis A^40,41^, which matches our observations in the here tested cell cultures. These somewhat controversial results obtained from different cell lines support the view that conclusions from a single cell line cannot be generalised and should be in the context of the corresponding phenotype. Importantly, the same epitopes can be present on different glycan classes (including *N*-glycans, *O*-glycans, and glycosphingolipid) and play different roles, showing the added value of a glycan class-specific phenotypic characterisation by e.g. mass spectrometry.

#### *N*-acetylhexosamine content

Another *N-*glycan feature, which was strikingly different between the investigated cell lines, was the higher or equal number of HexNAc than Hex of various *N-*glycans. Based on the loading plot of the PCA this feature was most dominant in PaTu-S cells (Fig. 4D). Derived trait analysis showed indeed an increase of these structures in PaTu-S cells (ø 27% HexNAc = Hex and ø 29% HexNAc > Hex) in comparison to PaTu-T (ø 11% and ø 10%), PDAC1 (ø 9% and ø 10%), PDAC2 (ø 17% and ø 18%), and hTERT-HPNE (ø 5% and ø 3%; Supplemental Table S2, Fig. 3I,J). The higher or equal number of HexNAc than Hex represents *N-*glycans with terminal HexNAcs and includes glycoforms with agalactosylated antennae, bisecting *N-*acetylglucosamine (GlcNAc), or attachment of *N-*acetylgalactosamine (GalNAc) residues in so-called LacdiNAc (LDN) structures as well as blood group A epitopes (GalNAc-[Fuc]-Gal-R) or SdA antigen (NeuAc-[GalNAc]-Gal-GlcNAc-R). Accordingly, galactosylation was lowest in PaTu-S (ø 85%), whereas hTERT-HPNE was characterised by almost complete galactosylation of complex type *N-*glycans (ø 99%; Supplemental Table S2). Fragmentation of the previously described *N*-glycan Hex4HexNAc5dHex3 at *m/z* 2142.78 (Fig. 2B) by MALDI-TOF/TOF-MS/MS gave neither indicative ions for bisecting GlcNAc nor the presence of a LDN residue. For few others, the indicative fragment ion for LacdiNAc (at *m/z* 429.1) was found (Supplemental Table S1), but often the position of the additional HexNAc could not be determined with certainty using MALDI-TOF/TOF-MS/MS fragmentation and additional LC-ESI-MS/MS experiments on AA-labelled *N*-glycans were performed. These experiments identified *N*-glycans containing LDN epitopes as shown for the glycan Hex3HexNAc6Fuc3 with indicative fragment ions at *m/z* 407.3 [HexNAc2 + H]^+^ and *m/z* 553 [HexNAc2Fuc1 + H]^+^ observed in LC-MS/MS spectra (Fig. 2C). A fragment ion of composition [HexNAc2Fuc2 + H]^+^ is likely caused by glycan rearrangement upon mass spectrometric fragmentation^42^ and may therefore not be seen as a proof of di-fucosylated antennae structures. The presence of fucosylated LDN structures (LDNF) was further confirmed with a monoclonal antibody against this epitope by flow cytometry, demonstrating its expression in PaTu-S cells (ø MFI 124.1), while expression on the other PDAC cells was (almost) absent and only low in hTERT-HPNE (ø MFI 11.5; Fig. 6G). In addition, we previously demonstrated the increased expression of blood group A epitopes containing GalNAc residues in PaTu-S compared to PaTu-T^38^. We further observed terminal HexNAc residues in a number of colorectal cancer cell lines^18^, and Satomaa *et al*. reported about abnormal non-reducing terminal GlcNAc residues on protein- as well as lipid-linked glycans in lung cancer^43^, indicating tumour-associated incomplete glycan synthesis. Other reports demonstrated that B4GALNT3-promoted LDN expression enhanced malignant phenotypes of colon cancer cells^44^, while suppressing neuroblastoma cell migration and invasion^45^. Overall, studies reporting the occurrence of LDN(F) epitopes and their involvement in cancer progression are scarce, and further investigations are necessary to increase our understanding about their functions.

#### Antennarity

Di-antennary *N-*glycans, defined as compositions with HexNAc = 4, were lowest expressed in PaTu-S (ø 24%) and increased in the more metastatic PaTu-T (ø 48%), PDAC1 (ø 46%), PDAC2 (ø 50%) as well as in the control pancreatic cell line (ø 58%, Supplemental Table S2, Fig. 3K). In contrast, *N-*glycans with HexNAc = 6 show the reversed trend (Fig. 3L) representing tetra-antennary structures, but also *N-*glycans containing HexNAcs in different constitutions such as LacNAc-repeats or GalNAc additions, thereby skewing this trait and making conclusions on antennarity challenging. Increased tri- and tetra-antennary glycans, however, have been previously associated with cancer progression in pancreatic cancer cell lines and serum of patients with PDAC^46^.

Strikingly, PaTu-S cells, which show the more epithelial tumour-like behaviour, differed most strongly from the normal epithelial-like pancreatic duct cell line with regard to complex-type *N-*glycan features, whereas their high-mannose type content was comparable and lower than for the more metastatic cells. PaTu-T and PDAC1 showed similarities in various *N-*glycan features such as HexNAc content and multi-fucosylation, while PaTu-T and the control cell line hTERT-HPNE showed most *N*-glycomic similarities and shared for example high levels of α2,3-sialylation and similar expression patterns of (sialyl) Lewis antigens. Clearly, functional studies with more cell lines as well as in-depth studies on the role of the individual glycan motifs in these model systems are needed to elucidate the meaning of these diverse (*N*-)glycan profiles in the process of metastasis in PDAC.

## Conclusion

The here presented *N-*glycomic characterisation of different PDAC cells revealed major differences as compared to the *N*-glycome of a control normal pancreatic cell line, suggesting changes of the *N*-glycosylation in PDAC. However, we also found pronounced different *N-*glycan profiles between the four PDAC cells, which did not directly relate to their functional behaviour. These data suggest not only different mechanisms of cancer progression and metastasis by the model systems studied, but also show the diversity of model systems. The latter raises the concern about choosing the right model system as well as drawing conclusions and generalising results from only one or few cell lines. PDAC2 cells, for example, were characterised by high expression of Lewis B and Y, (sialyl) Lewis A and sialyl Lewis X and showed high metastatic potential *in vivo* in zebrafish, whereas the PaTu-S cells shared high levels of sialyl Lewis A and Lewis Y antigens and were least invasive. Sialyl Lewis A is used as diagnostic marker (CA19-9) in PDAC and was lowest expressed in the control cell line, but also the metastatic cell line PaTu-T did not show high expression of this antigen. Furthermore, the normal pancreatic duct cell line shared high levels of sialyl Lewis X with PDAC2, demonstrating that further studies on different glycan-based mechanism involved in PDAC metastasis formation are needed and that different model systems may lead to different conclusions. Moreover, the results of this study challenge the genuinely normal character of the control cell line hTERT-HPNE and raise concerns as to what extent transfections (not only in this case) have unwanted effects on the geno- and phenotype of the cells – making proper characterisations essential.

To improve the quality and reliability of cell lines as (glycobiological) model systems, it is essential to characterise the model system and link it to the investigated disease/cancer phenotype. Mass spectrometry offers a great tool for the glycomic characterisation in an untargeted and glycan-type specific manner, while data from direct binding studies with anti-glycan antibodies and lectins by flow cytometry supported and refined the mass spectrometry-based results. The use of lectins and antibodies gave further an insight in the overall surface glycosylation, while the mass spectrometric characterisation of other glycan classes is still needed and ongoing.

Importantly, discrepancies between results obtained from different cell lines demonstrate that findings from one cell line cannot be generalised for the biology of a cancer. Experiments with higher numbers of cell lines as well as functional analysis of individual glycan motifs within these model systems are needed to improve the impact of these studies.

## Materials and Methods

### Materials

HPLC SupraGradient acetonitrile (ACN) was obtained from Biosolve (Valkenswaard, The Netherlands) and dithiothreitol (DTT), ethanol, sodium bicarbonate (NaHCO_3_), and glacial acetic acid were from Merck (Darmstadt, Germany). Ammonium formiate, dimethyl sulfoxide (DMSO), 8 M guanidine hydrochloride (GuHCl), 1-hydroxybenzotriazole (HOBt) hydrate, 2-aminobenzoic acid (AA), 2-picoline borane, 50% sodium hydroxide (NaOH), trifluoroacetic acid (TFA), and super DHB matrix (2-hydroxy-5-methoxy-benzoic acid and 2,5-Dihydroxybenzoic acid, 1:9) were obtained from Sigma-Aldrich (Steinheim, Germany) and 1-ethyl-3-(3-dimethylaminopropyl)carbodiimide (EDC) from Fluorochem (Hadfield, UK). *N-*Glycosidase F (PNGase F) was purchased from Roche Diagnostics (Mannheim, Germany), and the peptide calibration standard was purchased from Bruker Daltonics (Bremen, Germany). MultiScreen HTS 96 multiwell plates (pore size 0.45μm) with high protein-binding membrane (hydrophobic Immobilon-P PVDF membrane) were purchased from Millipore (Amsterdam, The Netherlands), 96-well polypropylene 0.8 mL 96-deepwell plate and 96-well PCR plate polypropylene from Greiner Bio (Alphen a/d Rijn, The Netherlands). Control Visucon-F plasma pool from 20 healthy human donors (citrated and 0.02 M HEPES buffered) was obtained from Affinity Biologicals (Ancaster, Canada). All buffers were prepared using Milli-Q water (mQ) generated from a Q-Gard 2 system (Millipore).

### Cells and cell culture

Pa-Tu-8988S and Pa-Tu-8988T pancreatic cell lines were purchased at the DSMZ culture bank (Braunschweig, Germany). PDAC1 and PDAC2 primary cell cultures were isolated from patients at the University Hospital of Pisa (Pisa, Italy) as described in Avan *et al*.^22^, and human pancreatic duct epithelial-like cells hTERT-HPNE (also referred to as normal or control cell line) was acquired from the American Type Culture Collection (ATCC, Manassas, VA). An overview of cell characteristics is given in Table 1. Cell culturing was performed as described previously^15,17^ and primary PDAC cells were kept at low passage number (<30).

**Table 1:** Overview of pancreatic cells and characteristics. Information was obtained from DKMS, ATCC and given literature.

Cell Line	Cell Type	Origin	Special	Morphology
PaTu-S	Pancreas adeno-carcinoma (PDAC)	Liver metastasis of a primary PDAC from a 64-year-old female^*^	Sister cell line of Pa-Tu-8988T	Epithelial-like phenotype, polarity, E-cadherin expression, preserved cell-cell contacts, low migratory properties^51^.
PaTu-T	PDAC	Liver metastasis of a primary PDAC from a 64-year-old female*	Sister cell line of Pa-Tu-8988S	Mesenchymal-like phenotype, spindle-like, increased cell projections, no E-cadherin expression, minimal cell-cell contacts, highly migratory both *in vitro* and *in vivo* ^51^.
PDAC1	PDAC	69-year-old male^22^	Primary cell culture	Epithelial-like
PDAC2	PDAC	64-year-old female^22^	Primary cell culture	Less cohesive pattern of growth with irregular cytoplasmic borders
hTERT-HPNE	Pancreas, duct	Intermediary cells formed during acinar-to-ductal metaplasia, 52-year-old male	Normal	Epithelial-like

*Same liver metastasis.

### Sample preparation and MALDI-TOF-MS

*N-*glycans were released from eight biological replicates per cell line (0.5 × 10 E6 cells each in four (batch A) or two (batch B) technical replicates randomly distributed across the plate) using a 96-well plate PVDF-membrane based *N-*glycan release protocol followed by linkage-specific sialic acid derivatisation, purification by cotton-HILIC-SPE, and MALDI-TOF-MS analysis as described earlier^18^. As controls, Visucon pooled human plasma as well as water blanks were used.

### Data processing of MALDI-TOF-MS spectra

Spectra were processed as described earlier^18^. Shortly, a composition list was generated based on smoothed (Savitzky Golay algorithm, peak width: *m*/*z* 0.06, 4 cycles), baseline corrected (Tophat algorithm), and internally re-calibrated average spectra using FlexAnalysis 3.4 (Stable Build 76) and the open-source software mMass (http://www.mmass.org)^47^. Compositional annotations as well as MS/MS peak annotations were performed in GlycoWorkbench 2.1 stable build 146 (http://www.eurocarbdb.org/) using the Glyco-Peakfinder tool (http://www.eurocarbdb.org/ms-tools/). Calibration (see Supplemental Table S4) and targeted data extraction of individual mass spectra was achieved with our in-house software developed for automated data processing, MassyTools version 0.1.8.0^48^. Spectrum as well as analyte quality was assessed using several quality parameters calculated within the software. Finally, the intensities of revised analytes were rescaled to a total relative intensity of 100% for each spectrum (which passed the quality control). The curated data is available as Supplemental Table S5.

### Data analysis

Rstudio statistical software environment (Version 0.99.892, http://www.r-project.org/) was used to remove batch-effects using the sva library for ComBat Batch correction. Furthermore, derived traits such as galactosylation, fucosylation, sialylation, etc. were calculated in Rstudio by applying in-house developed scripts combined with open-source packages (Calculations see Supplemental Table S6). Outliers were excluded and technical replicates were averaged for each biological replicate for direct as well as derived glycan traits. Differences between the cell lines with regard to glycan classes (derived traits) were analysed performing a principal component analysis (PCA) in SIMCA (Version 13.0; Umetrics AB, Umea, Sweden). Unit variance (UV)-scaling was applied to the data and validation of the model was performed by internal cross-validation (CV) based on biological replicates (n = 8) as CV groups.

Relative abundances of derived traits were visualised in boxplots using the ggplot2 library in Rstudio. Additionally, observations from the PCA were confirmed by Mann-Whitney statistical test performed in Rstudio on derived traits with adjustment of the significance level for multiple testing and *p*-values < 0.00037 were considered significant (Supplemental Table S7).

### LC-ESI-ion trap-MS/MS of AA-labelled *N-*glycans

Remaining released *N-*glycans were pooled per cell line (PaTu-S, PaTu-T and hTERT-HPNE) and labelled using an equal volume of freshly prepared labelling solution (48 mg/mL AA in DMSO/15% glacial acetic acid and 1 M 2-picoline borane in DMSO, 1:1 *v/v*) and an incubation for 2 h at 65 °C^49^. Samples were cooled down to room temperature, brought to 85% ACN and purified by HILIC-SPE as described previously^18^.

Released, AA-labelled, and purified *N-*glycans (5 µL sample) were then injected into a nano-RP-LC-ESI-ion trap-MS(/MS) system consisting of an Ultimate 3000 RSLCnano system (Thermo Scientific, Sunnyvale, CA) coupled to an ESI nano sprayer (Bruker Daltonics). Samples were loaded on a trap column (Acclaim PepMap100 C18 column, 100 μm × 2 cm, C18 particle size 5 μm, pore size 100 Å, Thermo Scientific) for concentration prior to separation on an Acclaim PepMap RSLC nano-column (75 μm × 15 cm, C18 particle size 2 μm, pore size 100 Å, Thermo Scientific). A flow rate of 500 nL/min was applied in a multistep linear gradient (t = 0 min, c(B) = 1%; t = 5 min, c(B) = 1%; t = 20 min, c(B) = 25%; t = 25 min, c(B) = 70%; t = 30 min, c(B) = 70%; t = 31 min, c(B) = 1%; t = 55 min, c(B) = 1%) with 0.1% formic acid in water as solvent A and 0.1% formic acid in 95% ACN and 5% water as solvent B.

Mass spectrometry was performed on an AmazonSpeed ion trap (Bruker Daltonics) in positive ion mode with a mass window of *m*/*z* 400 to *m*/*z* 2000 for MS analyses. Selected precursor masses (see Supplemental Table S8) were further analysed in MS/MS mode with ion detection over *m*/*z* 100 to *m*/*z* 2800. Fused-silica capillaries with an internal diameter of 20 μm were used for electrospray (1300 V) and solvent evaporation was achieved at 220 °C with a stream of nitrogen at a flow rate of 3 L/min.

### Flow cytometry analysis

Flow cytometry was performed as described in Belo *et al*.^38^. Briefly, pancreatic cancer and normal cells were incubated for 1 h at 37 °C in TSM (20 mM Tris-HCl, pH 7.4, 150 mM NaCl, 1 mM CaCl2 and 2 mM MgCl2), biotinylated plant lectins (10 µg/ml) *Sambucus nigra agglutinin* (SNA) and *Maackia amurensis agglutinin* (MAA) (E-Y Laboratories; San Mateo, CA) or at 4 °C with antibodies (5–10 μg/mL) against the glycan antigens Lewis X (clone P12), Lewis Y (clone F3), Lewis A (clone unknown) and Lewis B (clone T218) (Calbiochem, Darmstadt, Germany), sialyl Lewis A (LS-B5366, LifeSpan Bioscience, Seattle, WA), sialyl (dimeric) Lewis X (clone FH6; Biolegend, San Diego, CA) and against the antigen LDNF^50^, kindly provided by Dr. Richard Cummings (Harvard Medical School, Boston, MA). Next, cells underwent a 30 min staining with a fluorescent secondary antibody (goat anti-rabbit Alexa Fluor 488, goat anti-mouse Alexa Fluor 647, anti-mouse IgM Alexa Fluor 647; Molecular Probes, Invitrogen, Carlsbad, CA), using cells stained only with these secondary antibodies as background fluorescence negative controls. Binding of lectins and anti-glycan antibodies was measured using a FACSCalibur or a BD LSRFortessa™ flow cytometer (both BD Biosciences, San Jose, CA). FlowJo v10.3 (FlowJo, LLC, Ashland, OR) and Summit software (BD Biosciences) were used to determine cell population mean fluorescence intensity (MFI). Averages of measurements from a minimum of three independent experiments were calculated.

## Electronic supplementary material

Supplemental Tables S1-S8
